# The Week's Work

**Published:** 1910-04-30

**Authors:** 


### The Week's Work.

### COVENTRY HOSPITAL ACCOMMODATION FOR DIPHTHERIA.

The Coventry Sanitary Committee has just issued a report on the utility of the local hospital accommodation for diphtheria cases, and investigation into the subject has been thoroughly carried out by Dr. Snell, the medical officer of health for the district. Dr. Snell reports that at present there is no accommodation provided for such cases in the City Isolation Hospital, serious cases requiring tracheotomy being admitted to the Coventry and Warwickshire Hospital wards. The committee points out that there are two special reasons why hospital accommodation for diphtheria patients is particularly desirable—firstly, because bad cases cannot very well be treated at home; and, secondly, on account of the community, isolation in diphtheria cases being particularly necessary. These reasons are well known and ought to have appealed to the Coventry Council long ago, for the city has never been free from the disease. Last year's statistics are not yet available, but in 1908 some 108 cases were notified, with eight deaths. Coventry, however, is not singular in providing no accommodation for cases of the disease, for a similar state of affairs prevails at Aston Manor, Walsall, West Bromwich, Wigan, Wolverhampton, Handsworth, South Shields, and Sunderland. It is to be hoped that the report of the medical officer of health will cause the Coventry authorities to embark upon an isolation ward scheme. A hospital for at least 20 patients is necessary, and, as the medical officer of health points out, if such a ward were made an adjunct to the City Hospital, and tracheotomy cases were admitted, provision should be made for a resident medical officer on the premises, and definite quarters would have to be arranged for him in the administrative block.

### HOSPITAL FARMING.

When the House of Lords Committee on the hospitals and the nursing question took evidence some years ago, one or two of the witnesses were asked if their institutions, which possessed country estates, had made any attempt to utilise these properties as farms in order to supply their hospitals with garden produce. In two cases, we believe, the experiment was actually made, but was soon dropped as the working expenses were found to be altogether out of proportion to the benefits derived from the scheme. Since then, however, we have advanced in matters concerning farming, and it is now possible, as has been shown by one or two American institutions, to farm a few acres so successfully that they may be depended upon to supply the institution that owns them with all the necessary vegetables and "garden sass." In most hospital reports there is an item "Gardens," under which heading are included paths and approaches, window decoration and general horticultural arrangements, no matter how small they are. The garden as a productive piece of work does not exist in the majority of institutions. At some of the sanatoria, notably, we believe, at Frimley, the garden is a feature in which the patients as well as the staff take a great deal of interest, and of which they are justifiably proud. In the Continental hospitals it is the invariable rule that convalescent patients should spend some part of their day in garden work if they are fit to do so, and the mild labour given to them is usually much appreciated, while the institution gains in more ways than one by adopting this plan. Usually, however, gardens of this type are purely ornamental; they are not productive, and cannot be depended upon to yield contributions to the steward's department. It yet remains a question if it is not possible for institutions advantageously to farm their own produce. At present, of course, the supply of meat, in the shape of beef and mutton, is beyond the means of even an institutional trust; but it is possible that some day in the future such a trust or co-operative hospital society may find it worth while to have its own dairy farms in the Argentine or in Rhodesia. Similarly a hospital trawler may in the future be considered an indispensable outlay for any institution that desires to be up to date. While, however, these are hopes and aspirations for the future, the realisation of which demands much careful thought and mutual help, the provision of small farms on hospital-owned estates is another matter which may reasonably engage the attention of institutional workers at present. The chief question here is whether or not the hospital can, by working such corners of its estates, obtain sufficient produce to obviate its buying fresh vegetables by contract. If this can be done at a cheaper rate than is possible by tendering, the matter ought to be seriously considered, for in that way the hospital can be absolutely certain of obtaining good sterling stuff, while it saves any middleman's profit.

### AN EXPERIMENTAL FARM WANTED.

The question has not been satisfactorily solved by the experiments made at the time of the Lords Committee. These experiments were not definite, but rather sporadic, and led to no result because they were not properly thought out. The initial cost of starting an experimental hospital farm is likely to frighten governors, but it is just as well to bear in mind that at the present time, when agricultural land is depreciating in value and the income of hospitals from country estates is steadily falling, it may be better financial policy to work such estates on behalf of the hospitals themselves than to let them out at low rentals. A system of co-operative farming might be tried, the institution helping a tenant farmer and guaranteeing the acceptance of a certain quantity of produce at fixed charges. Where the produce in question consists of the more commonly used vegetables, the quantities required of which are well known or can be accurately estimated every day, it would be comparatively easy to arrange for a constant supply. In the case of fluctuating and varying quantities, where the supply must be regulated in accordance with the institutional demands, an attempt to provide such produce in a manner likely to be satisfactory to farmer and hospital will hardly be successful. This is especially true of poultry, and one hospital alone will find it exceedingly bad policy to venture on a poultry farm. Taking all factors into consideration, self-help in the matter of farming is a problem which the hospitals who possess the necessary land and can afford the initial outlay would do well to attempt to solve. It has already engaged the attention of one or two smaller institutions in America, and in a future issue we hope to give a *résumé* of what has been done in this direction in other parts of the hospital world. Meanwhile we shall be glad of any information on the subject which hospital secretaries are able to furnish.

### MODERN OPERATING THEATRES.

In the April number of *Het Ziekenhuis* Dr. van Eysselsteyn criticises a recent editorial of ours dealing with the modern hospital operating theatre, an editorial which has also been commented upon by the German and American institutional press. As our readers may remember, we therein pointed out that there was a tendency, in our opinion not entirely justified by results, to ultra-refinement in the designing and equipping of modern operating theatres, leading to a great deal of unnecessary expense. We suggested that it would be worth while to gather statistical information regarding the results obtained by the workers in the most finished theatres of the strict aseptic type and those secured by surgeons working in rooms designed without such absolute regard to the old Breslau school. We did not point out, however, that there is a tendency on the part of many surgeons who have had long experience of the finnicky trivialities that obtained in the ultra-aseptic theatres to return to the older types, under the firm impression that absolute asepsis was impossible under existing conditions. This has been the case at Breslau, where the solemn routine—one may almost call it ritual—of the late Dr. Mickulicz has been much modified, to the lasting convenience and comfort of everyone attending operations at that excellent clinic, and with, so far as we have been able to gather, no increase in the septic percentage, or no untoward results as far as the operations themselves are concerned.

### A DUTCH AUTHORITY'S CRITICISM.

Dr. van Eysselsteyn recognises, in part, the justice of our views, but he objects, and perhaps rightly, to taking the results of statistics as definitely proving the superiority of any type of theatre over the others. The personal factor is the most important, and different surgeons working in the same theatre will have different results. "Without doubt," our critic goes on, "the results of operations will depend much more upon the operator than upon the theatre. The latter, apart from slovenliness and indifference, contributes only a very small percentage of the dangers which threaten the patient. Hence it is good that The Hospital should warn against exaggeration which may become pathological and hugely increase the expenses without producing corresponding compensatory results. An operator or assistant with a cold is much more dangerous to the patient than a simple light placed above the table so long as this is kept clean. Light obtained from without the theatre by means of reflectors is by no means ideal. Artificial ventilation with heated air led into the theatre by means of pipes that are difficult to keep clean is still less satisfactory. Shafts so arranged as to remove everything that is unclean from the room are decidedly wrong, since they become veritable breeding-places for infection through which air enters into the warmed theatre. One may multiply these objections, and anyone who lays himself out to do so will easily find opportunity to strain at gnats and swallow camels in order to increase the costs of building and equipment." At the same time it is imperative that strict simplicity and cleanliness be preserved in modern designs, and that provision should be made for proper and efficient ventilation, lighting, and cleansing.

### OUR POSITION.

While regretting "the retrogressive movement," of which he thinks our editorial supplies evidence, Dr. van Eysselsteyn considers that a protest against over-refinement may do good at the present time. With this view we certainly cordially agree. We voiced no retrogressive opinions in our article, and are not the supporters of a conservatism that preserves economy at the expense of thoroughness and efficiency; but we are convinced that a simpler type of operation-room model will be found to answer as well as the much more expensive and more complicated types in fashion in Germany, America, and some English hospitals. And while we agree that a statistical tabulation of results will prove comparatively little to the worker who has already investigated the subject of simplicity *versus* complication, we still think that such a tabulation will draw attention more prominently to the need for revision in our estimates when they are extravagant through the demands of some surgeons.

### THE DRUG MARKET.

The demand for drugs and chemicals has not been very active, and interest continues to be absorbed by transactions in rubber. There has been a slight reduction in the price of quicksilver, which may possibly be followed by a reduction of 1d. per lb. in the price of calomel and other mercurial salts, but this is not certain. Ipecacuanha seems to be tending cheaper, and there is a disinclination to buy at the present high prices. Buchu leaves are again dearer. Cod-liver oil is slightly cheaper. Castor oil has advanced in price considerably. The price of citric acid is tending higher. The value of opium is well maintained.

